# GenTIGS: a database empowering research and clinical insights on rare genetic disorders with an Indian perspective

**DOI:** 10.1093/nargab/lqaf143

**Published:** 2025-11-11

**Authors:** Iliyas Rashid, Pooja S, Shivranjani C Moharir, Rakesh K Mishra

**Affiliations:** Tata Institute for Genetics and Society, GKVK Post, Bellary Road, Bengaluru 560065, India; Tata Institute for Genetics and Society, GKVK Post, Bellary Road, Bengaluru 560065, India; Tata Institute for Genetics and Society, GKVK Post, Bellary Road, Bengaluru 560065, India; Academy of Scientific and Innovative Research (AcSIR), Ghaziabad 201002, India; Tata Institute for Genetics and Society, GKVK Post, Bellary Road, Bengaluru 560065, India; Academy of Scientific and Innovative Research (AcSIR), Ghaziabad 201002, India

## Abstract

Rare genetic disorders (RGDs) are conditions affecting fewer than 1 in 2000 individuals. Recent advances in genetics and healthcare renewed hope for better diagnosis and treatment. RGDs are common in India due to consanguinity and limited diagnostic testing. To converge the global- and India-specific knowledge about RGDs to a single platform for researchers, clinicians, and stakeholders, we developed GenTIGS database—a “go-to platform” for information retrieval and data analysis. GenTIGS is a comprehensive database containing information about genes, pathogenic variants, and clinical symptoms, with a focus on disorders reported globally and in India. GenTIGS delivers a variety of features and data points important to researchers, academicians, and clinicians in the RGD domain, ensuring efficient information retrieval. This platform provides information on 2306 RGDs and 2772 associated genes, including 691 disorders reported in India. It also includes details on 3525 clinical symptoms and 307 340 pathogenic variants for these disorders. GenTIGS offers comprehensive information and analytical tools for in-depth analysis and exploration of genes and variants associated with RGDs, thus supporting progress in genetic medicine and research through enhanced understanding and analysis. Accessible at https://db.tigs.res.in/gentigs/.

## Introduction

Rare genetic disorders (RGDs), stemming from gene mutations or function-altering variations, present unique challenges due to their infrequent occurrence and consequential impact on affected individuals [1, 2]. RGDs are classified as monogenic or polygenic based on the involvement of genes [3]. Monogenic disorders are usually associated with mutations in a single gene at a time [4]. Such mutations are referred to as pathogenic genetic variants, and these are mostly germline mutations that follow a Mendelian inheritance pattern [5]. Genetic heterogeneity [6] is a characteristic of monogenic disorders, which involves either different mutations in the same gene in an individual case, such as the GLA gene mutation in Fabry disease [7], or mutations that arise one at a time in several diverse genes, each of which can cause the same disorder such as Usher syndrome [8]. Unlike monogenic disorders, simultaneous mutations in multiple genes cause polygenic disorders, which follow environmental factors and do not follow Mendelian inheritance mode [9]. In polygenic disorders, the effect of each gene mutation is minimal, and the mutations in multiple genes collectively produce complex traits [3]. Monogenic RGDs can exhibit dominant or recessive inheritance patterns and can be either autosomal or sex-linked [10].

Often, chronic and severely disabling conditions impose a substantial burden on healthcare systems [11, 12]. The World Health Organization reports that over 6000 RGDs affect 1 in 2000 individuals, particularly in pediatric conditions [13–15], accounting for 3.5%–5.9% of the world population, with some RGDs being rare in specific demographic groups [16, 17]. Of the >6000 rare diseases, 71.9% are genetic [18] and nearly 2150 RGDs have associated gene information available in RARe-SOURCE (Table 1). The realm of rare disorders historically lacked attention in the scientific community due to the lack of resources and technology. Though with the advent of sophisticated techniques like parallel sequencing, the landscape of research has shifted, leading to the diagnosis and reports of new rare diseases and conditions [19]. While some disorders have attracted great attention from healthcare workers, the field remains in its initial stages [20], with challenges in tracing lineage in affected families and obtaining sufficient DNA samples from different generations for analysis [21]. The International Rare Diseases Research Consortium (IRDiRC) perspective reveals that many genes and variants associated with RGDs are still unknown, highlighting the need to accelerate discovery efforts [22].

**Table 1.** Full names of abbreviated resources useful for analyzing genetic disorders

Resource abbreviation	Full resource name	Access link
ClinGen	Clinical Genome Resource	https://clinicalgenome.org/
ClinVar	Clinical Variation Database	https://www.ncbi.nlm.nih.gov/clinvar/
EMBL-EBI OLS	European Bioinformatics Institute Ontology Lookup Service	https://www.ebi.ac.uk/ols4
GARD	Genetic and Rare Disease Information Center	https://rarediseases.info.nih.gov/
gnomAD	Genome Aggregation Database	https://gnomad.broadinstitute.org/
GRDR	Global Rare Diseases Patient Registry Data Repository	https://grdr.hms.harvard.edu
GTR	Genetic Testing Registry	https://www.ncbi.nlm.nih.gov/gtr/
GUaRDIAN	Genomics for Understanding Rare Diseases: India Alliance Network	https://guardian.genomes.in/
HPO	Human Phenotype Ontology	https://hpo.jax.org/
IRDiRC	International Rare Diseases Research Consortium	https://irdirc.org/
MedGen	Medical Genetics Database	https://www.ncbi.nlm.nih.gov/medgen/
NCBI	National Center for Biotechnology Information	https://www.ncbi.nlm.nih.gov/
NIDAN Kendra	National Inherited Disorders Administration Kendras	https://dbtindia.gov.in/dbt-press/inauguration-nidan-kendras-and-ummid-launch-dbt-website
NORD	National Organization for Rare Disorders	https://rarediseases.org/rare-diseases/
NPRD	National Policy for Rare Diseases	https://rarediseases.mohfw.gov.in/
OMIM	Online Mendelian Inheritance in Man	https://www.omim.org/
RARe-SOURCE™	Details on genes associated with rare diseases	https://raresource.nih.gov/
UMMID	Unique Methods of Management of Inherited Disorders	https://dbtindia.gov.in/scientific-directorates/information-systems-biology/ummid

The cases of RGDs are burdened in India due to lack of awareness, endogamy, and limited access to genetic testing [23]. These burdens are compounded by financial constraints, social stigma, and inadequate healthcare infrastructure [24]. Research initiatives on RGDs in India are focused on developing novel diagnostics and discovering novel treatments by applying clinical trials and advanced genetic sequencing technologies in collaborative networks [25, 26]. Personalized treatment strategies and specific gene therapies offer a potential solution to improve the quality of life of people with rare genetic conditions [27, 28]. In India, patient advocacy groups, nonprofit organizations, and government initiatives work together to raise awareness through research promotion, policy changes, and public awareness efforts [29, 30].

Several resources and databases provide globally reported information on RGDs, helping users to access and interpret data on various aspects of diagnosis, treatment, and research (Table 1). Some resources cover human genetic disorders and provide detailed information on clinical features, inheritance patterns, genetic mutations, associated genes, and related research [18, 31]. Numerous databases have been developed through collaboration among researchers, healthcare professionals, and advocacy groups to provide essential information on genetic disorders and patient care services [32–34]. NCBI resources [35] offer a set of databases on genetic disorders such as gene data, disease association studies, genetic testing, and clinical variant interpretation. In addition, several resources and dashboards accelerate diagnosis and treatment by standardizing, classifying, and interpreting RGD information [36–39].

India is focusing on enhancing services and data management related to genetic variations and rare conditions in the Indian population to address them in research, diagnosis, and treatment strategies [40–42]. Identification of population-specific variants in >2800 samples collected from 260 South Asian cohorts showed promising potential in reducing the disease burden [43]. India’s health priorities have changed in recent years, with an increased focus on noncommunicable diseases, and there has been significant progress in RGD research [44]. Initiatives including the GUaRDIAN Consortium [45], NPRD 2021, UMMID, and NIDAN Kendra (Table 1) address clinicians and researchers for understanding, prevention and control, early detection, treatment, genetic counseling, and specialized care of RGDs in India [46]. Despite multiple data delivery systems on genetic disorders in the world, very few efforts have been made to manage RGD-related genes and other associated information on a centralized platform in the Indian context. Such a platform will play a vital role in integrating data to analyze the clinical status of genetically challenging conditions reported in the Indian population to improve early diagnosis, treatment, and research. A well-organized database on RGD and related information will promote research collaboration and information sharing. This will help healthcare institutions, researchers, and policymakers to understand genetic patterns and mutations in individuals living with and without any clinical conditions and symptoms.

In India, where access to basic healthcare remains a significant challenge, over 72 million people are affected by rare diseases [47], further straining an already burdened healthcare system [48]. In this context, an initiative such as GenTIGS supports clinical research by consolidating diverse information on a single platform to enhance the understanding of the treatment of RGDs, helpful to reduce the strain. Though there are many databases on rare diseases in the world, mainly focused on patient care services. Here, an attempt has been made to collect and store information on only those RGDs for which the corresponding gene and mutation data were available. GenTIGS is focusing on monogenic RGDs reported in India, which are isolated and identified based on case reports and research articles and integrated with a dedicated task-oriented interface for browsing information. It bridges the knowledge gap about RGDs by providing genetic information for each disorder as well as other related information, making it valuable for research and effectiveness in the undeclared conditions.

## Materials and methods

### Database creation

The database development, data curation, and query management were executed using Linux, Apache, MySQL, PHP, and Perl (LAMPP). A relational database management system, MySQL, was utilized to structure tables for diverse data management at the backend, running on the Linux operating platform. The database was designed with multiple tables and applied bidirectional normalization to rows and columns to eliminate redundancies. Additionally, indexing was implemented across several tables to optimize query retrieval times.

### Collection, curation, and population of data in the database tables

**Figure 1.**
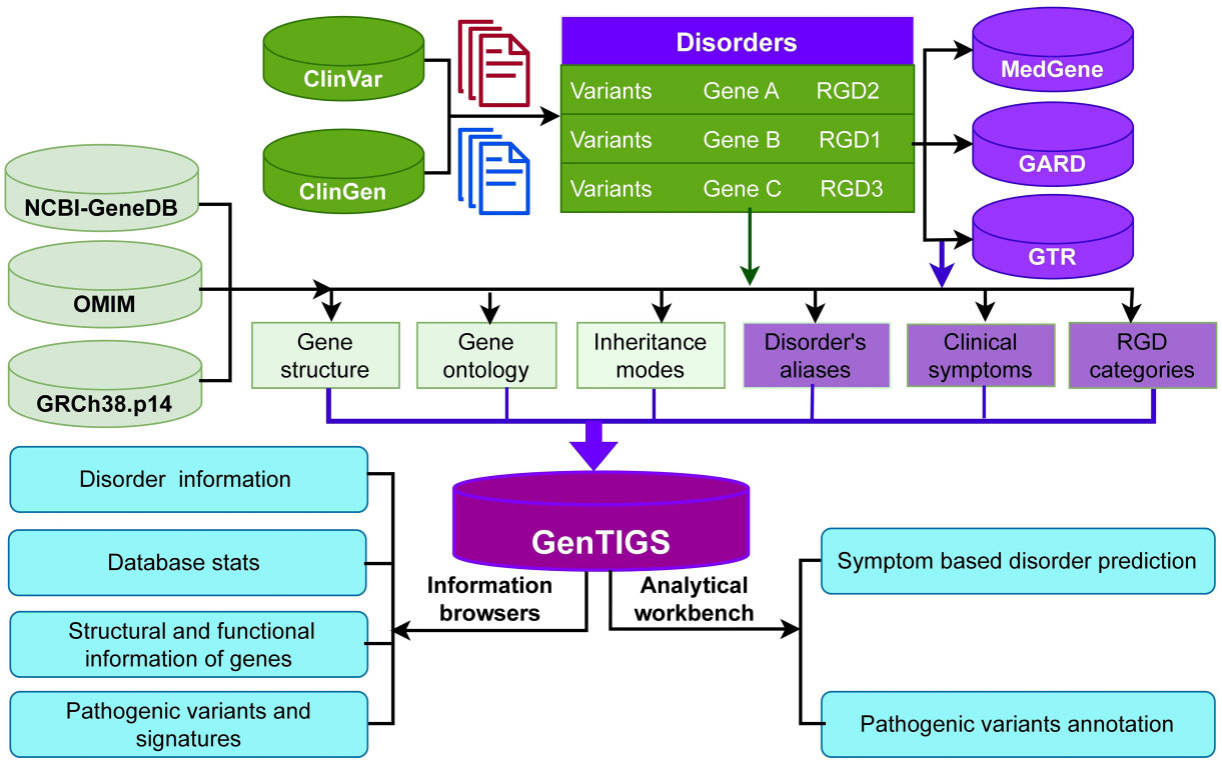
Architecture and data flow diagram of GenTIGS. The schematic outlines the processes of collecting, segregating, enriching, and curating information on rare genetic disorders from multiple data sources, with the final output accessible via user-friendly web interfaces for browsing and analysis.

We utilized NCBI resources [35] as the primary data source for compiling and integrating information into RGDs. Our approach involved gathering comprehensive data on RGDs and their associated genes through an exhaustive search. Following the initial data collection, we enhanced our dataset by incorporating additional curated information from various reliable sources. Data collection was initiated by compiling a list of RGDs and their associated gene names from case reports and research articles using PubMed [35]. In the next process, disorders detected in India were segregated from the broader list based on case reports and research articles specifically reflecting disorders reported in India. To further enrich the information of the listed RGDs, we sourced responsible variants for each disorder from ClinVar [49] and ClinGen [50]. To enhance the depth of information, we also gathered inheritance modes, disorder categories, clinical symptoms, aliases, and relevant annotations from OMIM [51], NCBI-MedGen [52], GARD [33], and GTR [53]. Detailed gene information, sequences, and associated annotations were retrieved from NCBI-Gene [54] and human genome GRCh38.p14 [55]. MalaCards [38] was also used to obtain supplementary data, disorder subcategories and classifications, and integrated with our listed RGDs. A dataflow diagram illustrates the collection and curation of datasets from various resources for integration and management in the database, providing different webpages for browsing and analysis (Fig. 1). All datasets and annotations were curated according to the schema of the respective tables of GenTIGS and managed accordingly by writing Perl programs. Several in-house Perl programs were developed using the database interface module for database connection, schema design, table definition, and data management. The individual program addressed specific tasks, including data curation, mapping, comparison, and integration of relevant datasets obtained from various sources, and populating them into respective tables. Additionally, Perl scripts such as “NCBIGeneDB_Mapper.pl” and “GeneSeqParse.pl” [56] were employed for parsing gene information and sequences and managing them. Finally, GenTIGS data delivery system was incorporated with efficient data browsing and analysis facilities.

### GenTIGS database update pipeline

GenTIGS is updated routinely. For database updates, the first step is a manual search for case reports and research articles on RGDs that are not already in GenTIGS using appropriate keywords. Manual curation is applied to maintain accuracy and relevance in the database. This approach excludes disorders that are cited only for reference or as examples in case reports or research articles of other disorders or experiments performed on cell lines to investigate disorders. As per the flow diagram (Fig. 1), the names of the listed RGDs are used by in-house programs to map corresponding data sources, parse them, and finally store them in the database. We repeat the same process for each update by reviewing recent publications and adding newly reported RGDs along with the corresponding gene information in the database.

### Metadata analysis

Variant call format (VCF) files from the IndiGenomes project, which includes 1029 self-reported healthy males and females over the age of 30 from diverse regions across India [57], were made publicly available. These files were subsequently downloaded and processed using a custom script, “IndiGenMapper.pl,” to identify pathogenic variants associated with autosomal dominant and recessive inheritance patterns in this cohort of self-declared healthy individuals. This script aligns variants from VCF files with ClinVar data in the GenTIGS database, focusing on genes associated with disorders reported in India. The custom script categorized mapped variants as “identical” (exact match of mutational positions and alleles) or “similar” (same mutation site, different genotypes), as exemplified in Table 2. The mapped variants were further classified into “pathogenic with conflicts” and “pathogenic without conflicts” using ClinVar annotation and organized into the database. The Venny tool [58] was used to visualize the frequencies of identical and similar variants, categorized as pathogenic or nonpathogenic. In this analysis, variants reported in the GRCh38.p14 genome from ClinVar data were considered for annotation, as IndiGenomes used the same reference genome assembly.

**Table 2.** Representation of identical and similar mapped variants

Example of identical mapped variants	Similar mapped
• IndiGen: chr13:51944170:C>T	• IndiGen: chr10:89247612:C>T
• ClinVar: chr13:51944170:C>T	• ClinVar: chr10:89247612:AC>A
• ClinVar Entry: NM_000053.4(ATP7B):c.3182G>A (p.Gly1061Glu)	• ClinVar Entry: NM_000235.4(LIPA):c.37del (p.Val13fs)
• Condition: Wilson disease (AR)	• Condition: Wolman disease (AR)
• Exact match of mutational positions and alleles	• Same mutation site, different genotypes

Presenting a detailed comparison of clinically significant variants identified in two datasets: IndiGen and ClinVar. Variants are classified as identical or similar based on their genomic positions, allele changes, and corresponding clinical data.

### Web Interface

For web-based information delivery and analysis, user interactive dynamic web pages were designed and implemented using LAMPP, CGI (common gateway interface), Ajax, JavaScript, CSS (cascading style sheets), and HTML technologies.

## Results

This database is exclusively dedicated to storing information on RGDs and their associated genes, as reported globally, along with a segregated list of disorders that are documented in India based on case reports. The GenTIGS web interface offers user-friendly menu items such as “Home,” “Disorders,” and “Analysis.” Apart from these disease-specific menus, it also has “Contact,” “Feedback,” and “Keyword search” options that further facilitate the ease of use and make the interface more interactive. The web-based workbench of GenTIGS includes the “Disorders” and “Analysis” menu items for retrieving, browsing, and analyzing gene information associated with an RGD. Continuous updates are implemented as new information emerges. It serves as a user-friendly search and data delivery system, simplifying data retrieval and facilitating data analysis of monogenic disorders.

### Home menu

**Figure 2.**
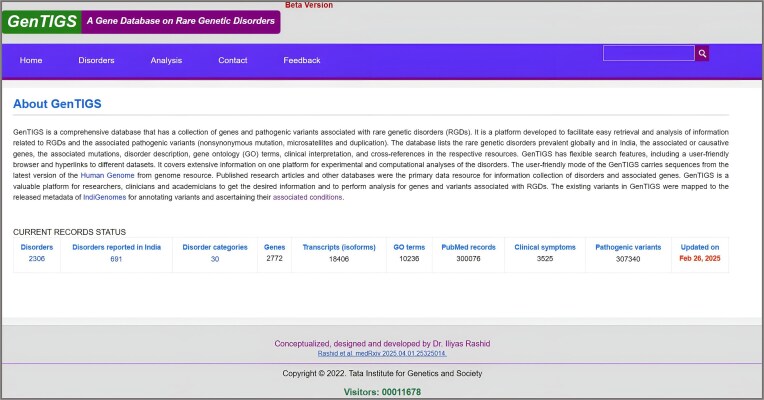
GenTIGS data delivery system. Displays statistics of RGD reported globally and in India, and information on their inheritance mode, associated genes, gene ontology, pathogenic variants, and clinical symptoms.

The homepage provides an overview of the database and displays statistics with the latest updates. It gives information about the purpose served by the database, how and from where the content is curated, and the significance of the database. GenTIGS currently includes 2306 globally reported RGDs associated with 2772 genes, of which 691 RGDs are known to occur in India across 30 major disorder categories linked to 3525 clinical symptoms and influenced by 307 340 pathogenic variants (Fig. 2). It also includes information on aliases, inheritance modes, and subcategories of RGDs. A detailed breakdown of the total subcategories and RGD-associated genes across various categories, along with their association with single or multiple genes, is provided in Table 3.

**Table 3.** Major disorder categories

Category name	Total subcategory	Total RGD	RGD-associated genes	
			Single genes	Multiple genes
Aging disorders	0	2	2	0
Blood disorders	11	34	18	16
Bone disorders	13	91	61	30
Cancer disorders	25	40	16	24
Cardiovascular disorders	7	14	4	10
Developmental/cancer	0	1	0	1
Developmental/multisystemic disorders	0	3	2	1
Ear disorders	1	2	0	2
Ear disorders/hair disorders	0	1	1	0
Endocrine disorders	8	18	12	6
Eye disorders	7	26	14	12
Eye disorders/ear disorders	1	3	0	3
Gastrointestinal disorders	7	11	5	6
Hair disorders	0	2	1	1
Immune disorders	7	34	24	10
Liver disorders	1	3	1	2
Metabolic disorders	18	112	77	35
Metabolic disorders/lysosomal storage disorders	8	31	21	10
Multisystemic disorders	1	17	12	5
Nephrological disorders	9	20	7	13
Neurodegenerative disorders	19	50	36	14
Neurodevelopmental disorders	9	36	19	17
Neuromuscular disorders	6	56	35	21
Neuronal disorders	3	7	6	1
Oral disorders	3	6	5	1
Pain syndrome	0	1	0	1
Reproductive disorders	2	2	1	1
Respiratory disorders	8	11	4	7
Skin disorders	21	54	38	16
Tumor/cancer	0	3	1	2
30	195	691	423	268

Summary of subcategories, total RGDs, and single- versus multigene-associated disorders across major disorder categories.

### Disorder menu

The “Disorders” menu in GenTIGS offers two flexible dropdown options: (i) “Disorders reported in India,” which focuses on data of Indian-reported RGDs and (ii) “Worldwide disorders,” which provides a global perspective. These options allow for efficient access to RGD information, catering to both regional and global disorder data on RGDs.

#### Disorders reported in India

The “Disorders reported in India” section functions as the RGD summary webpage, serving as the central information hub within GenTIGS for those RGDs that are reported in India. This module facilitates viewing consolidated key information presented in multiple tables on the same page. It retrieves and displays different types of information about the associated RGDs by selecting the RGD name from the built-in RGD selection list at the top right of the page. The retrieved content includes the RGD and its aliases, major disorder categories, Mendelian inheritance patterns, and associated genes. The RGD name and alias are displayed at the top of the disorder summary webpage, with the inheritance mode and disorder category below, hyperlinked to their respective webpages for more detailed information. The RGD summary webpage also provides a detailed description of the associated gene(s), including the gene name, location of the gene on the strand, number of exons, chromosomal information, cytogenetic bands, and genomic accession in the top central table. The last column of the table links users to external gene databases, such as OMIM, gnomAD Browser, Ensembl, GeneCards, and ClinGen, for easy navigation and access to cross-database gene information. A “More” hyperlink in the gene information table leads to the “gene details” page, which reveals information on messenger RNA, transcript variants, and isoforms. These entries are linked to PubMed IDs referencing articles where each isoform has been reported. A four-tier graphical representation was developed for the visual interpretation of the selected gene mapped on the chromosome, all exons, and reported pathogenic variants, based on their positional coordinates and strand orientation. Additionally, 500 bp consecutive segments, which collectively cover the entire length of the gene, are presented in a table showing the number of pathogenic variants in each segment. Those consecutive segments were visualized in a histogram next to the segment table. Another table in “gene details” displays the variants identified in the same gene as part of the IndiGenomes resource, a large-scale genomic initiative from India, which includes 1029 genome samples from adult males and females across the nation. Additionally, the gene detail page features another table representing Gene Ontology (GO) annotations.

The “RGD summary” page under “Disorders reported in India” features a central table displaying variant information, showing the total number of variants identified in genes associated with the selected disorder. This table includes a selection list of clinically significant variants, including pathogenic variants, which enables users to explore information of selected clinically significant variants reported in genes linked to the chosen RGD. The “Disorders reported in India” section includes a table at the bottom, showing clinical symptoms, medical terminology, descriptions, and the occurrence frequencies associated with selected RGD. Furthermore, symptoms linked with a specific frequency type for the selected disorder can be viewed in the table by selecting the frequency type from the built-in dropdown selection list.

In addition to detailed and referenced information, each disorder was redirected via hyperlinks to several widely used databases, such as Orphanet, OMIM, GARD, GTR, MedGen, EMBL-EBI OLS, MalaCards, Monarch Initiative, Human Phenotype Ontology, and NORD. These links facilitate GenTIGS data validation and provide global access to comprehensive datasets from each database, offering diverse and specialized information.

#### Globally reported disorders

The “Worldwide disorders” is accessible under the “Disorders” menu in GenTIGS. It provides information on disorders and their associated genes reported from various regions around the world, including India. While offering a global perspective, it serves as a superset of the “Disorders reported in India.” The integrated web interface retrieves and displays information of the user-selected RGD name from an in-built selection list. The displayed information includes the name of the selected disorder and the name of the associated gene, and each entry is cross-referenced to well-known databases. RGDs reported across the world are valuable resources of GenTIGS for researchers, clinicians, and patients exploring knowledge about monogenic disorders. Analysis of disease-causing genetic factors will help to determine what contributes to the common and unique features of a clinical condition in different populations.

### Analyzing information

The “Analysis” menu has tools for symptom-based disease prediction and metadata analysis that enhance the utility and visibility of the database (Fig. 2). The “Analysis” menu has two dropdown items: (i) “symptom-based disorder prediction” predicts RGDs linked with specific traits and (ii) “metadata analysis” provides an analysis overview of variants in Indian samples. These features enable access to disorder data and analytical results, support in-depth research, and display various genetic variants for better insight into genetic conditions.

#### Disorder prediction interface

The symptom-based disorder prediction tool is accessible through “Analysis” dropdown menu item in the user interface. A flexible user-friendly query interface is provided, offering a concise description and a manual outlining the steps for initiating disorder predictions. The interface includes a category selection list, which allows users to choose a specific category of interest or to select all categories. Upon selection, the corresponding clinical symptoms associated with the chosen category (or all categories) are displayed in a list on the left-hand side of the selection box. Users can then select at least one or more clinical symptoms. Once clinical symptoms are selected, users can initiate the disorder prediction process by clicking the “Predict RGD” button. The predicted disorder(s) and relevant information are then presented dynamically below the query area in the same interface window, providing an immediate and interactive result display.

The RGD prediction displays the top 10 predicted disorders in a table “Top predicted RGDs,” set in descending order by score and alphabetically if the disorders had the same score. Numerical weights of disorder prediction were calculated by quantifying the frequency of each symptom associated with a disorder. Each frequency value is standardized, such that “always” is given a weightage of 4, indicating a strong relationship; “very frequently” and “frequently” are given weightages of 3 and 2, respectively; “sometimes,” “unusually,” and “rarely” are given low weightages of 1 and 0.5; and no frequency is given a weightage of zero. The weight scores support decision-making in predicting disorders and diagnosing clinical conditions. On the “Disorder prediction” page, a box of selected symptoms related to the predicted disorders appears to the right of the “Top predicted RGDs” table. The names of the disorders and disorder categories, the medical terms related to the symptoms, their frequency of occurrence, and a description of the symptoms are displayed in another table at the bottom of this page. The displayed names of the disorders are hyperlinked, directing users to the GenTIGS Central page “Disorders reported in India” for detailed information.

#### Variant annotations

We developed a program that queries metadata from human population genomes to identify pathogenic variants and their related conditions. This tool was applied to the recently released IndiGenomes dataset that includes genomes sequenced from 1029 individuals. The tool facilitated annotating the variants and associating them with the different conditions reported in GenTIGS. The results of this analysis are accessible via GenTIGS platform and can be retrieved through the “IndiGenomes metadata” option in the “Analysis” menu of the user interface. This interface features a dropdown list of various clinical significances for identically mapped variants to retrieve and display information in a centralized table. Identifiers of samples displayed in the table are hyperlinked and directed to IndiGenomes variant pages to view valuable data such as genotype, allele count, or frequency.

**Figure 3.**
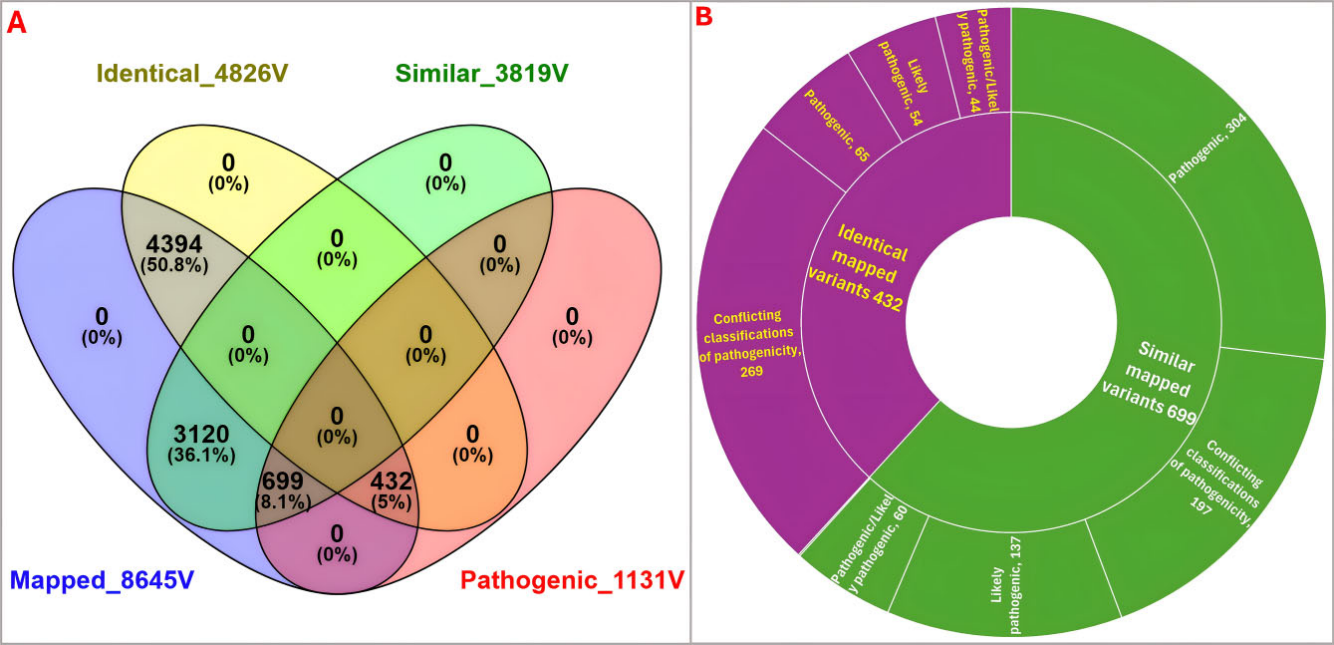
Annotation of IndiGenomes variants. (**A**) The Venn diagram illustrates the overlap of clinically significant variants in GenTIGS and IndiGenome across four datasets—identical, similar, mapped, and pathogenic variant sets. (**B**) The sunburst chart further classifies the 1131 clinically significant variants into the following categories: pathogenic, likely pathogenic, and variants with conflicting pathogenicity.

During variant annotation and mapping of the IndiGenomes VCF file onto ClinVar in the GenTIGS database for disorders reported in India, two types of mappings were observed. The first involved “identical variants,” in which the position and mutation were exactly the same in both datasets. The second type consisted of “similar variants,” where the position was identical but the variant type and mutational base pairs differed. The synonymous mutations, unless reported pathogenic (and hence identical), were categorized into similar variants group. The integration of the IndiGenomes dataset with the GenTIGS database led to the identification of 8645 variants, including 4826 identical mutation sites, 3819 similar mutation sites, and 1131 pathogenic variants, which encompass both identical and similar mutations (Fig. 3A). Among these, 1131 distinct pathogenic sites were categorized into 432 identically mapped variants and 699 similar mapped variants (Fig. 3A). A more detailed analysis of these pathogenic variants, categorized by clinical significance, is shown in Fig. 3B. A total of 432 pathogenic variants were identically mapped and assigned phenotypes and Mendelian inheritance modes. Every identically mapped variant can be accessed using the above-described “IndiGenomes metadata” section under the “Analysis” menu.

An in-depth analysis of the 65 identical pathogenic variants depicted in Fig. 3B identified 14 dominant phenotypes (Table 4) and 37 recessive phenotypes (Table 5) of RGDs, all of which have been reported in India. The remaining pathogenic variants were related to X-linked and multimode inheritance patterns. Furthermore, all clinically important variants, both pathogenic and benign, were added to the database for reference and analysis. The variants with similar mapping in Fig. 3 are listed with detailed information on their clinical significance in Supplementary data. These findings highlight the importance of precise mapping in identifying exact mutations in clinical genomics.

**Table 4.** Annotation of variants linked to autosomal dominant disorders

IndiGen_VCFid	ClinVar variants	ClinVarID	Related disorders
chr7:143332723:G>T	chr7:143332723:G>T	2950953	Congenital myotonia, autosomal dominant form (AD)
chr2:165309366:T>C	chr2:165309366:T>C	976279	Episodic ataxia (AD)
chr16:23629212:C>A	chr16:23629212:C>A	2 062838	Familial cancer of breast (AD)
chr17:43071239:C>G	chr17:43071239:C>G	441389	Hereditary breast ovarian cancer syndrome (AD)
chr1:45331503:G>A	chr1:45331503:G>A	406824	Hereditary breast ovarian cancer syndrome (AD)
chr7:117665565:G>A	chr7:117665565:G>A	53929	Hereditary pancreatitis (AD)
chr22:26026799:G>A	chr22:26026799:G>A	2506003	Klippel–Feil syndrome (AD)
chr11:17396980:C>T	chr11:17396980:C>T	9098	Leucine-induced hypoglycemia (AD)
chr22:20994728:G>C	chr22:20994728:G>C	984443	Noonan syndrome (AD)
chr16:375373:C>A	chr16:3757373:C>A	158358	Rubinstein–Taybi syndrome due to CREBBP mutations (AD)
chr2:165991694:G>A	chr2:165991694:G>A	1048587	Severe myoclonic epilepsy in infancy (AD)
chr14:91314114:G>A	chr14:91314114:G>A	2841702	Spinocerebellar ataxia type 40 (AD)
chr1:209788590:G>A	chr1:209788590:G>A	458682	Van der Woude syndrome 1 (AD) and Popliteal pterygium syndrome (AD)
chr13:77903200:G>A	chr13:77903200:G>A	16639	Waardenburg syndrome (AD)

This table lists annotated pathogenic variants from the IndiGenomes VCF along with their association to dominant disorders reported in India through GenTIGS labeling.

**Table 5.** Annotation of variants linked to autosomal recessive disorders

IndiGen_VCFid	ClinVar variants	ClinVarID	Related disorders
chr16:88810517:G>A	chr16:88810517:G>A	988047	Adenine phosphoribosyltransferase deficiency (AR)
chr9:452071:C>T	chr9:452071:C>T	2868856	Autosomal recessive hyper-IgE syndrome (AR)
chr15:42410958:G>C	chr15:42410958:G>C	195641	Autosomal recessive limb-girdle muscular dystrophy type 2A (AR)
chr11:66511012:G>T	chr11:66511012:G>T	551439	Bardet–Biedl syndrome (AR)
chr12:88049337:A>T	chr12:88049337:A>T	1071318	Bardet–Biedl syndrome (AR) and nephronophthisis (AR)
chr1:230236716:C>T	chr1:230236716:C>T	873546	Congenital disorder of glycosylation (AR)
chr16:68691842:T>G	chr16:68691842:T>G	800872	Congenital hypotrichosis with juvenile macular dystrophy (AR)
chr7:50470090:G>A	chr7:50470090:G>A	2976771	Deficiency of aromatic-l-amino-acid decarboxylase (AR)
chr2:232480717:C>A	chr2:232480717:C>A	1048794	Distal arthrogryposis type 5D (AR)
chr19:35848087:C>T	chr19:35848087:C>T	56439	Finnish congenital nephrotic syndrome (AR)
chr14:87939926:A>T	chr14:87939926:A>T	370392	Galactosylceramide beta-galactosidase deficiency (AR)
chr1:155239937:G>A	chr1:155239937:G>A	1321421	Gaucher disease (AR)
chr19:12893515:T>C	chr19:12893515:T>C	3015902	Glutaric aciduria, type 1 (AR)
chr17:42911016:G>A	chr17:42911016:G>A	961497	Glycogen storage disease due to glucose-6-phosphatase deficiency type IA (AR)
chr3:33068940:C>T	chr3:33068940:C>T	264673	GM1 gangliosidosis type 2
chr9:10142754:C>T	chr9:101429754:C>T	218380	Hereditary fructosuria (AR)
chr1:156875009:G>A	chr1:156875009:G>A	1323379	Hereditary insensitivity to pain with anhidrosis (AR)
chr13:101073631:G>A	chr13:101073631:G>A	684708	Hypotonia, infantile, with psychomotor retardation and characteristic facies 1 (AR)
chr5:14812 905:C>T	chr5:148123905:C>T	959534	Ichthyosis linearis circumflexa (AR)
chr6:129314798:G>A	chr6:129314798:G>A	2735188	LAMA2-related muscular dystrophy (AR)
chr1:68439640:T>A	chr1:68439640:T>A	2949504	Leber congenital amaurosis (AR)
chr12:88055666:A>AT	chr12:88055666:A>AT	156386	Meckel syndrome, type 4 (AR)
chr6:129316041:G>T	chr6:129316041:G>T	948070	Merosin deficient congenital muscular dystrophy (AR)
chr1:197090004:G>A	chr1:197090004:G>A	157918	Microcephaly 5, primary, autosomal recessive (AR)
chr8:89971175:G>A	chr8:89971175:G>A	2792660	Microcephaly, normal intelligence and immunodeficiency (AR)
chr12:101749181:G>A	chr12:101749181:G>A	38429	Mucolipidosis type II (AR)
chr16:88822614:C>T	chr16:88822614:C>T	2823297	Mucopolysaccharidosis, MPS-IV-A (AR)
chr9:131512069:C>T	chr9:131512069:C>T	598282	Muscular dystrophy-dystroglycanopathy (congenital with brain and eye anomalies), type A1 (AR)
chr12:88071890:T>TA	chr12:88071890:T>TA	545704	Nephronophthisis (AR)
chr15:89321782:G>T	chr15:89321782:G>T	2843494	Progressive sclerosing poliodystrophy (AR)
chr17:47945558:G>A	chr17:47945558:G>A	6524	Pyridoxal phosphate-responsive seizures (AR)
chr2:71515752:G>A	chr2:71515752:G>A	290198	Qualitative or quantitative defects of dysferlin (AR)
chr1:94000900:G>A	chr1:94000900:G>A	99458	Severe early-childhood-onset retinal dystrophy (AR)
chr6:33173902:G>A	chr6:33173902:G>A	1455992	Otospondylomegaepiphyseal dysplasia (AR)
chr1:215970661:T>TTGGACTCTGAAGGAATG	chr1:215970661:T>TTGGACTCTGAAGGAATG	956100	Usher syndrome (AR)
chr13:51944170:C>T	chr13:51944170:C>T	312383	Wilson disease (AR)
chr2:31373894:T>TG	chr2:31373894:T>TG	2073968	Xanthinuria type II (AR)

This table lists annotated pathogenic variants from the IndiGenomes VCF along with their association to recessive disorders reported in India through GenTIGS labeling.

### Keyword search

This interface includes a powerful keyword search feature that enables keyword searching by various terms such as disorder name, alias, gene name, gene symbol, and gene ID to retrieve appropriate records from the database. This interface provides multiple views to display relevant information depending on the type of search keyword entered by the user. These different views are designed to ensure that the information retrieved is clear and organized, allowing easy access to the details associated with each keyword. If the keyword searched by the viewer is unique, either the full name of a gene or a disorder, the search will lead to the RGD main page and display information related to RGD. If the keyword search is a partial or common name reflecting multiple disorders or gene names present in the database, the search browser displays a table listing hyperlinked RGDs with summarized information, each further leads to the RGD main page.

## Discussion

The diversity of genetic conditions in India, including the prevalence of inherited genetic diseases across different populations, necessitates the resources that account for regional and ethnic variations [48]. As highlighted by the India State-Level Disease Burden Initiative collaborators [59], India’s vast ethnic diversity and the presence of founder mutations in some populations demand a comprehensive genetic resource along with real-world data for in-depth analysis [57]. Such databases provide insights into both the local and global contexts of genetic disorders and their detailed genetic information, bridging the gap in understanding and diagnosis of RGDs in the Indian setting [45, 60]. By integrating data from major international repositories, alongside region-specific information, this approach is particularly useful for studies and clinical applications for regional as well as global perspectives [61, 62]. GenTIGS was developed through the integration of multiple data sources, including data on RGDs that had been documented in India, to improve the comprehensiveness of the database within this context. This facilitates an in-depth exploration of RGDs with a global focus, while also addressing the unique genetic landscape of India. Lagunes-García proposed a method for collecting diverse disease information from public sources and their cross-referencing to create unified resources with multiple data points [63]. The disorders included in GenTIGS are based on multiple data points gathered from reputable sources. Linking that information to external resources enhances data reliability and allows researchers to cross-validate findings with other databases, thereby ensuring the quality and accuracy of the information. The detailed coverage of multiple information on RGDs helps users to understand epidemiology and genetics associated with conditions. Importantly, the inclusion of clinical symptoms and their frequencies further enhances the practical utility of the platform for clinicians, enabling better diagnosis and management of RGDs [33]. In the context of family information management related to inherited conditions [64], the inclusion of inheritance modes associated with each disorder in GenTIGS allows users to analyze genetic diseases based on their transmission patterns.

The development of enriched web services for biomedical data visualization [65], clinical decision support systems [66], and interactive tools for meta-analysis of diagnostic test accuracy [67] has had a significantly advanced role in healthcare. Such services assist in improving data analysis, graphical interpretation, and decision-making. The implemented features and customized search functions of GenTIGS make it possible to search for information related to disorders, genes, aliases, and identifiers. This platform ensures quick and accurate data retrieval of targeted information, which is useful for both clinical and research applications. Digital polymerase chain reaction (dPCR) is used for the important screening of pathogenic variants in genes for the diagnosis of childhood-onset genetic disorders [68]. The Gene information section displays a view of 500 bp contiguous fragments covering variations and position coordinates across the gene. This module can be helpful for efficient screening of newborns in cohorts or at the population level by selecting the segment targeting specific variants to design dPCR primers and amplification. Additionally, the disorder predictions and variant annotation applications present systematic results and facilitate easy interpretation of the data.

GenTIGS expands research and improves understanding in diagnosis and personalized medicine by leveraging customized modules in genetics and regional information. Such developments aid genetic counseling predominantly in regions affected by founder effects [69]. Its “Symptom-based disorder prediction” feature aids clinicians in predicting potential RGDs from clinical symptoms, streamlining diagnostics. The platform also integrates metadata analysis tools for annotating genetic variants, such as those from the IndiGenomes dataset, improving understanding of genetic diseases in the Indian population. This supports genotype–phenotype correlation studies to refine diagnostic precision and disease comprehension [70, 71].

The inclusion of the IndiGenomes dataset, which is derived from over 1000 healthy Indian genomes, provides a unique opportunity to study the genetic diversity of the Indian population and its implications for RGDs. We utilized GenTIGS to perform a genome-wide comparative analysis of the IndiGenomes released dataset. The comparative analysis revealed a set of identically mapped pathogenic variants associated with both autosomal and recessive modes of genetic disorders reported from different parts of the world. Recessive disorder is caused when both copies of a gene need to carry the associated pathogenic variant for the manifestation of the disease phenotype. If only one copy carries the variant, symptoms typically do not manifest in clinical conditions, though the individual is a carrier [72, 73]. A dominant disorder is caused when only one copy of the allele carrying the pathogenic variant (received from either parent) is sufficient for the disorder to develop. These conditions are linked to specific clinical outcomes, which may manifest in infancy or later in life, with varying severity [74, 75, 51]. In the present study, we identified the variants in the IndiGenomes dataset that were already reported in ClinVar and had dbSNP identifiers, beginning with the “rs” prefix like “rs762183907”. The pathogenic mutations in genes such as *MUTYH, FANCD2, NBN, PALB2*, and *BRCA1* are associated with hereditary breast and ovarian cancer syndrome, an autosomal dominant disorder typically manifesting around age 30 and above [76]. Some of these pathogenic variants can sometimes lead to conditions that manifest later in life. Some variants identified in this study were associated with familial and infantile disorders. However, the individuals carrying these variants remained healthy into adulthood. For example, the biallelic variant chr22:20994728:G>C reported in the IndiGen dataset is a pathogenic mutation LZTR1:c.1785+1G>C that causes Noonan syndrome 10. Noonan syndrome is generally considered an autosomal dominant condition, but recent studies suggest that the *LZTR1* gene may be implicated in both dominant and recessive forms of the disorder [77, 78]. Similarly, Waardenburg syndrome (EDNRB) and Van der Woude syndrome 1 (IRF6) have also been reported in autosomal dominant modes. This highlights that variants that are pathogenic in one context may be benign in another due to the interplay of genetic and environmental factors [79]. As participants in the IndiGenomes study were healthy individuals, these variants may represent benign polymorphisms or variants of uncertain clinical significance, suggesting that they do not directly contribute to the disease phenotype in this population.

The varying effects of genetic variants in different populations reflect discrepancies like allele frequencies, genetic background, and other factors. Studies show that healthy individuals carry pathogenic variants that have already been observed in patients, potentially due to factors such as gene dosage sensitivity [80], functional effect of the mutation [81, 82], heterozygous carrier status [83], and other genetic modifiers [84]. The IndiGenomes dataset revealed several dominant (Table 4) and recessive (Table 5) traits associated with pathogenic and other clinically significant variants (Supplementary data) in healthy individuals. In these cases, the identified variants may not cause the disorder due to insufficient disruption or a weak dominant effect. Thus, IndiGenomes emphasizes the important role of population-specific genomic data for understanding RGDs, as prevalence and mutation spectrum may vary among different ethnic groups [85].

In this study, we have developed methodologies for interpreting and analyzing data in the Indian context, but these concepts might be adapted for broader applicability. Previously, standardized national initiatives for variant curation, pathogenicity scoring, and inheritance annotation [86], and methods of ethnically diverse studies [45, 57] have already been used in global studies.

## Conclusion

GenTIGS is a versatile platform for RGD research and clinical genomics that stores diverse data on genetic and clinical traits integrated with analytical tools, especially in the Indian context. Alignment of GenTIGS with global databases enhances its reliability and applications in variant pathogenicity annotation, and disorder prediction reflects the potential understanding of RGD. Like other online databases, GenTIGS is also updated regularly, with upcoming reports and real-time information covering various aspects of RGD. In the coming days, analytical capabilities will be expanded, and clinical data will be added for its effective application. Like other methods and applications, the analysis module of GenTIGS also has some limitations. “Symptom-based disorder prediction” uses only variables as RGD-associated symptom frequency to evaluate weight matrices for predicting disorders. Integration of clinical and metadata for multivariable inputs and future expansion in performance model evaluation metrics will improve prediction efficiency, precision, recall, and accuracy in disorder prediction. Similarly, the partial VCF file available for public download can be used for restricted IndiGenomes metadata analysis. Lack of sample information in VCF is limiting allele frequency estimation during analysis of pathogenicity and variant annotation. Population-wide genomic data analysis, multi-omics data for deeper insights, and multivariate-based predictive models will enhance GenTIGS’ ability to find differences by explaining environmental conditions. A pathogenic variant in one population may be benign in another population, affecting the value of customized genetic assessments. Since the pathogenicity effects of variants found in Indian genomes in our comparative analysis may not be the same as those found globally. Inclusion of clinical case studies from diverse population areas like India will further elucidate the genotype–phenotype correlations and enhance its global significance. GenTIGS may have weight and impact in the Indian subcontinent, where genetic factors are likely to be more strongly correlated.

## Supplementary Material

lqaf143_Supplemental_File

## Data Availability

The GenTIGS database is openly accessible at https://db.tigs.res.in/gentigs/.
